# Authors' reply: Effect of preoperative oral antibiotics in combination with mechanical bowel preparation on inflammatory response and short‐term outcomes following left‐sided colonic and rectal resections

**DOI:** 10.1002/bjs5.50286

**Published:** 2020-04-16

**Authors:** A. M. Golder, co‐authors

**Affiliations:** ^1^ Academic Unit of Surgery, University of Glasgow Level 2, New Lister Building, Glasgow Royal Infirmary Glasgow G31 2ER UK

We acknowledge Hartrick and colleagues for their interest in and comments regarding our study^1^. They correctly note that our study involved two longitudinal groups (control group before 2015 and test group after 2015). This was stated clearly in the Methods section and acknowledged in the limitations paragraph of the Discussion. This study was carried out after a change in routine clinical practice within our centre regarding the use of oral antibiotics and mechanical bowel preparation, and this potential limiting factor was therefore unavoidable. Despite this, both groups were from an entirely enhanced recovery after surgery era, which we hope would minimize other unmeasured variations in practice. Nonetheless, as with any similar form of study, we were unable to identify or account for all potential variables, hence our interest in ongoing RCTs within this field.

Hartrick *et al*. correctly note that a small proportion of patients in the test group had colorectal resection for benign disease, unlike the control group in which all resections were carried out for malignant disease. This was documented clearly in the Methods and Results sections, and also acknowledged in the limitations paragraph of the Discussion. For several reasons, we do not believe this introduced a significant bias to the results. The proportion of these patients was small (less than 15 per cent) and, although the authors have raised concerns regarding the possible heightened inflammatory state in malignant compared with benign disease, we propensity score‐matched for the preoperative systemic inflammatory response (modified Glasgow Prognostic Score) with good balance between test and control groups (Cramer's V = 0.018). By convention, *P* values for significance are not usually presented after matching, as the very fact that those variables have been used to generate the propensity scores leads to inherent bias and renders inference illogical. For this reason, Cramer's V was calculated before and after matching (*Tables 1* and *2* respectively), along with a ‘butterfly plot’ of propensity score distribution. Both methods showed improvement in balance after matching.

The authors correctly state that the C‐reactive protein and albumin cut‐offs for days 3 and 4 were the same as those used to calculate the postoperative Glasgow Prognostic Score on days 3 and 4. The outcomes included in *Table 3* are largely related – both the postoperative inflammatory state on postoperative days 3 and 4 and the development of postoperative complications^2^. As a result, the likelihood of a type I error is substantially less than it would have been had the study reported 15 unrelated outcomes. Regardless of statistical values, absolute numbers/percentages are shown in *Table 3* and are of clear clinical significance (a reduction in the overall complication rate from 55 to 28 per cent, a reduction in the infective complication rate from 37 to 20 per cent, and a reduction in the surgical‐site infection rate from 23 to 10 per cent). This was, however, a relatively small study with approximately 100 patients in each group, and clearly not powered to detect significant differences in less frequently observed complications including deep surgical‐site infections and anastomotic leaks.

As the above outcomes of interest are likely to be related, a Bonferroni correction is perhaps an overly conservative way of correcting for multiple testing. Given the interrelationship of our outcomes, a different analysis to correct for multiple testing such as the Benjamini–Hochberg procedure may be more appropriate^3^. Indeed, we have now carried out such a *post hoc* analysis. Using this correction, all of the outcomes reported as statistically significant in *Table 3* remained so when the false discovery rate was set at 5 per cent, and the majority remained statistically significant when the false discovery rate was sent at 10 per cent. Therefore, the suggestion of ‘*P*‐hacking’ is unlikely to be the case and is supportive of the peer review process.

Hartrick and colleagues state in their letter that the use of oral antibiotics and mechanical bowel preparation in resectional colorectal surgery is an important issue requiring further prospective research in the form of large prospective RCTs. As acknowledged in the final paragraph of the Discussion section of our article (‘This strategy is worthy of further investigation’), we are in clear agreement. Indeed, we look forward to the reporting of those trials currently underway, in particular the COLONPREP trial (EudraCT no. 2017‐002542‐72). This is of particular interest given the recent negative findings of the MOBILE trial^4^, contrary to most of the published meta‐analyses^5^, ^6^, ^7^ in the field.

## Disclosure

The authors declare no conflict of interest.
